# Congenital heart disease in men – birth characteristics and reproduction: a national cohort study

**DOI:** 10.1186/1471-2393-14-187

**Published:** 2014-06-02

**Authors:** Kristina Kernell, Gunilla Sydsjö, Marie Bladh, Niels Erik Nielsen, Ann Josefsson

**Affiliations:** 1 Department of Obstetrics and Gynaecology, Linköping University, Linköping, Sweden; 2 Department of Clinical and Experimental Medicine, Linköping University, Linköping, Sweden; 3 Department of Cardiology, Linköping University, Linköping, Sweden; 4 Department of Medical and Health Sciences, Linköping University, Linköping, Sweden; 5 Department of Obstetrics and Gynaecology, University Hospital, SE_581 85 Linköping, Sweden

**Keywords:** Congenital heart disease, Population register, Preconception care, Reproduction

## Abstract

**Background:**

Women with congenital heart disease (CHD) are more often born preterm or small-for-gestational age and with a caesarean section. This pattern together with an increased risk of congenital anomalies seems to be repeated in the next generation. Information on the effect of paternal CHD on their offspring is sparse. In this study we investigated if men with CHD differ from those who do not have CHD with respect to characteristics related to their own births, their reproductive patterns and the neonatal outcomes of their children.

**Methods:**

In this national cohort study data were derived from Swedish population-based registries. The population consists of all men born in 1973-1983 who were alive and living in Sweden at 13 years of age (n = 522 216). The index group is men with CHD (n = 2689). Men diagnosed with CHD were compared with men without CHD. The CHD were also divided into two groups, complex and simple CHD and comparisons between the groups were made.

**Results:**

Men with CHD are more likely to have been born preterm (p < 0.001), small-for gestational-age (p < 0.001) or large-for-gestational-age (p < 0.001) than men without CHD. They are also more likely to have been the result of a twin pregnancy (p < 0.001) and to have been delivered by caesarean section (p < 0.001). Men with CHD have a decreased likelihood to become fathers compared to non-CHD men and in this study their offspring do not have a higher incidence of CHD than offspring to non-CHD fathers. The neonatal outcomes of children of men with CHD do not differ from the outcomes of children of non-CHD men.

**Conclusions:**

Men with CHD were more often born with non-optimal characteristics compared to men without the condition. However, the increased risk does not repeat itself in the next generation. This knowledge can lead to improved preconception counselling for couples in which the father has a CHD.

## Background

During the past decades the percentage of people reaching adulthood who have congenital heart disease (CHD) has increased due to advances in medical and surgical procedures. CHD occurs in 0.5-0.8 percent of all live births and more than 85 percent of the group with CHD survive into adulthood [1,2]. Most probably, the majority of CHD men and women wish themselves to become parents.

In an earlier national cohort study we have shown that, women with CHD were more prone to give birth to children preterm or SGA and their babies were more often delivered by caesarean section with a higher frequency of congenital abnormality [3]. Other studies have also shown that CHD women experience a higher frequency of neonatal complications such as premature birth, small-for-gestational age birthweight, having children with congenital anomalies and a higher incidence of foetal and perinatal mortality than women without CHD [4-7].

The risk for the offspring to also have CHD is three to ten times higher in women with CHD than in women without [8,9]. Paternally-related CHD has been described in the literature, with reported risk of 2.5 to 3.6% [8,10,11].

Although we know much about the effect of CHD in the mother on outcomes, there is to our knowledge very little or no information about the effect of CHD in the father on outcomes of pregnancies to non-CHD mothers.

Swedish population-based registries contain well-validated data and are prospectively collected. These registries are mandatory and cover the entire population of 9 million people. They provide a unique opportunity to study the effect of CHD in men on the pre-, peri- and postnatal outcomes for their offspring born to non-CHD mothers.

The aim of our study was thus to investigate if men with CHD differed from those without CHD with respect to characteristics related to their own births, their reproductive patterns and the neonatal outcomes of their children.

## Methods

### Swedish registries

Data for this study were taken from Swedish population-based registries. Statistics of diseases and surgical treatment of patients in Sweden have been published for more than 100 years. With the Swedish personal identification number it is possible to link the data in the different registries to each other.

Since 1973 information concerning pregnancy, delivery and paediatric neonatal examination as well as certain maternal characteristics such as previous reproductive history has been collected in the Swedish Medical Birth Registry (MBR). Every health care provider is required to register birth information in MBR, which itself covers approximately 99 percent of all births in Sweden [12,13]. The Total Population Registry (TPR) contains data on births, deaths, marital status, as well as information on migration and country of origin for Swedish residents born abroad [14].

The National Patient Registry (NPR) has been in use since 1964 and from 1987 on it covers all public, in-patient care in Sweden [15]. The NPR continuously receives information on patient-, hospital-, and administration data, including diagnosis, external cause of injury, and surgical procedures. Since 2001 the register also contains outpatient visits including outpatient surgery and psychiatric care from both private and public caregivers. The diagnoses in the NPR are based on the Swedish version of the World Health Organization international classification of diseases (ICD). ICD-8 [16] was used until 1986, ICD-9 [17] was used between 1987 and 1996, and ICD-10 [18] from 1997 onwards.

The Causes of Death Register records information on all deceased persons registered in Sweden at the time of death [19].

By use of the Multi-Generation Registry (MGR) it is possible to identify the children of the men in the study population [20]. The children are also registered in the MBR and the TPR.

Information on the educational level of the parents of the men in the study population was retrieved from the Education Register and the Population and Housing Census 1970 [21,22].

Subjects with CHD were identified in this study as all for whom the records show one or more of the following three-digit-level codes: ICD-8: 746 or 747, ICD-9: 745-747, ICD-10: Q20-Q26. The men with CHD were found in the MBR and the NPR.

Two groups of CHD subjects were created, one “complex” and one “simple”. The main diagnoses leading to classification as “complex” are truncus arteriosus, transposition of the great arteries, tetralogy of Fallot and single-ventricle defects. The main diagnoses leading to classification as “simple” were isolated valve disease, isolated atrial or septal defect, coarctation of the aorta or persistent ductus arteriosus.

### Study population

All Swedish men born between 1973 and 1983 according to the MBR and the TPR who were alive and still living in Sweden at 13 years of age served as the study population (n = 522 216). The index group comprises men diagnosed with CHD at any time during life (n =2 689). The cohort was followed up until 2007. During the study period 4405 (0.84%) men in the total study population died of which a 272 also had become fathers. Using the MGR the firstborn children of the 522 216 men were found. The first birth occurred in 1989. A total of 110 419 father-firstborn-offspring pairs were identified. Due to missing values on birthweight or gestational length of the children 491 men were excluded in the calculations.

We retrieved information on the socioeconomic characteristics of the study group and their parents from the Population and Housing Census 1970 [23]. We also had information about the parents’ country of origin and their educational levels in 1985. Information on the mothers’ marital status, parity, and age at the time of giving birth (i.e. 1973-83) was also retrieved from the registries. We received information on several birth-related variables from records of the births of the men in the study group including birthweight, gestational length, instrumental delivery, caesarean section, as well as if the men were the result of a twin birth. The definitions of preterm birth and small-for gestational age (SGA) and large-for-gestational-age (LGA) follow those specified in the Swedish external standards from 1996 [24]. Preterm birth is defined as birth before 37 completed weeks of gestation, SGA as a birth weight < -2 standard deviations (SD) of the mean birth weight for the gestational length, and LGA age as a birth weight > +2 SD.

In the MGR the children of the men who have become fathers could be identified. The MBR and the NPR were used to obtain information about the children concerning birth weight, gestational length, delivery mode (instrumental or caesarean section), twin pregnancy and congenital malformations.

### Statistical analysis

Men diagnosed with CHD were compared to those not diagnosed with the condition by means of the χ^2^ – test. For continuous variables such as birth weight and gestational length comparisons between the groups complex CHD, simple CHD and non-CHD were made via analysis of variance corrected with Tukey-Kramer procedure. The data were also modelled through Cox’s proportional hazards model to estimate the effect of CHD on the men’s subsequent likelihood of becoming fathers during the study period. Age defined the time-dimension and the men exited from risk when they became fathers, died, or reached the end of follow-up, whichever took place first. Both crude and adjusted hazard ratios (HR) and corresponding 95% confidence intervals (CI) were calculated. Adjustments were made for the men’s parental socioeconomic characteristics, the age of the woman who gave birth to their child and whether this woman had a CHD.

In addition to the χ^2^ – tests, we performed a multivariable logistic regression analysis to estimate the differences between the men who were diagnosed with CHD and those not diagnosed with the condition, controlling for background variables such as the mothers’ and fathers’ educational levels, mothers’ marital status, mothers’ parity, parents’ country of origin and the different congenital heart malformations. A three level variable was created where 0 = no CHD, 1 = simple CHD and 2 = complex CHD. The odds ratios (OR) and corresponding confidence intervals (CI) were calculated and the groups were compared to each other. This enabled us to simultaneously account for the combined effect of the studied variables. However, as the differences between these analyses and the χ^2^ – tests presented in the tables were not substantial; we chose not to present the results of these additional analyses.

All analyses were performed using the IBM® SPSS® Statistics for Windows, Release. 18.0.1. 2009.

This study was approved by the Regional Ethical Review Board, Linköping, Sweden.

## Results

Of the men born in Sweden between 1973 and 1983 (n = 522 216), who were alive and still living in Sweden at 13 years of age, 2 689 (0.5%) had been diagnosed with CHD and 394 (14.7%) of these had complex CHD (Table 1).

**Table 1:** Distribution of congenital heart disease in Swedish men born 1973-83

**Diagnosis**	**Count**^**a**^
**Complex**	
Truncus arteriosus	25
Transposition of the great arteries	168
Tetralogy of Fallot	167
Single ventricle	34
**Simple**	
Ventricular septal defect	783
Atrial septal defect	534
Endocardial cushion-defect - atrioventricular septal defect	108
Triscupid valve defect	76
Mitral valve defect	135
Pulmonary valve defect	331
Congenital valve stenosis	244
Other heart defects	268
Congenital aortic insufficiency	332
Patent arterial duct	352
Coarctation of the aorta	265
Other anomalies of the aorta	103
Anomalies of the pulmonary artery	88
Anomalies of the great veins	119
Non-specified anomalies of the heart or great vessels	565
**Total**	**2689**

^a^Each person may have more than one CHD diagnosis.

In Table 2 the socioeconomic characteristics of the parents of the study population are presented. Mothers of men with a CHD were older when giving birth (p < 0.001) and more often unmarried (p = 0.047). There were no differences in the educational levels of the mothers or the fathers, in parity or country of origin. Table 3 shows the delivery related characteristics of the study group’s own births. Men with CHD were more likely to have been born preterm (p < 0.001), SGA (p < 0.001) and LGA (p < 0.001) than non-CHD men (Table 3). Men with CHD were also more often delivered by caesarean section (p < 0.001) and they were also more likely the result of a twin pregnancy (p < 0.001).

**Table 2:** **Socioeconomic characteristics of the parents of the studied men born in 1973-83**^a^

	**CHD**				
	**Yes**		**No**		
	**Count**	**%**	**Count**	**%**	**P-value**^**b**^
**Mothers’ educational level**					0.716
9-10 years	772	28.7	148226	28.5	
11-13 years	1108	41.2	218839	42.1	
> = 14 years	621	23.1	118229	22.8	
Missing	188	7.0	34233	6.6	
**Fathers’ educational level**					0.911
9-10 years	852	31.7	163384	31.4	
11-13 years	1081	40.2	207035	39.9	
> = 14 years	563	20.9	110334	21.2	
Missing	193	7.2	38774	7.5	
**Mothers’ marital status**					0.047
Married	1837	68.3	360903	69.5	
Divorced/widow	736	27.4	140602	27.1	
Unmarried	116	4.3	18022	3.5	
**Mothers’ age**					<0.001
13-19 years	97	3.6	20875	4.1	
20-26 years	1056	39.3	216352	41.6	
27-33 years	1129	42.0	221288	42.6	
> = 34 years	407	15.1	61010	11.7	
**Mothers’ parity**					0.142
No previous children	1101	40.9	220038	42.4	
Previous children	1588	59.1	299489	57.6	
**Parents’ country of origin**					0.788
One or both non-Nordic	202	7.5	39744	7.7	
Both Nordic	2487	92.5	479783	92.3	

^a^Analysis performed on all 522 216 men in the study cohort whose parents could be identified through the registries. All variables were measured at the time of the men’s birth (i.e. in 1973-83).

^b^Chi-squared test.

**Table 3:** **Birth characteristics of the men born in 1973-83**^**a**^

	**CHD**				
	**Yes**		**No**		
	**Count**	**%**	**Count**	**%**	**P-value**
**Birthweight (g) (mean/SD)**^**b**^	3328	720	3556	547	<0.001
**Gestational length (weeks) (mean/SD)**^**b**^	38.9	2.8	39.6	1.8	<0.001
**Born preterm**^**c**^					<0.001
Yes	339	12.6	26299	5.1	
No	2350	87.4	493228	94.9	
**Born small-for-gestational-age**^**c**^					<0.001
Yes	325	12.1	26213	5.0	
No	2364	87.9	493314	95.0	
**Born large-for-gestational-age**^**c**^					<0.001
Yes	158	5.9	22767	4.4	
No	2531	94.1	496760	95.6	
**Twin birth**^**c**^					<0.001
**Yes**	83	3.1	8230	1.6	
**No**	2606	96.9	511196	98.4	
**Instrumental delivery**^**c**^					0.8
Yes	179	6.7	35167	6.8	
No	2510	93.3	484360	93.2	
**Caesarean section**^**c**^					<0.001
Yes	437	16.3	51008	9.8	
No	2252	83.7	468519	90.2	

^a^Analysis performed on all 522 216 men in the study cohort.

^b^Analysis of variance corrected by Tukey-Kramer procedure.

^c^Chi-squared test.

There were 110 419 (21.1%) men in the study population who had become fathers during the study period. The proportion of men with different types of CHD who had become fathers is presented in Table 4. In Table 4 the p-values used for comparing men diagnosed with a specific CHD to all other men in the study are also presented and show that men with CHD have lower rates of paternity except for men with transposition of the great arteries, tetralogy of Fallot, single ventricle, triscupid valve defect, congenital valve stenosis, congenital aortic insufficiency, coarctation of the aorta and other anomalies of the aorta, anomalies of the pulmonary artery, and the great veins where no differences were found. Also, after adjustments for parental socioeconomic characteristics presented in Table 2, men with CHD were found to have a decreased likelihood of becoming fathers during the study period (HR = 0.767, 95% CI = 0.697-0.845, data not shown). Moreover, no difference in the incidence of paternity was found in the case of men with CHD having children with a woman with CHD compared to men with CHD having children with a woman without CHD; Hazards ratio = 0.210 (95% confidence interval 0.031-1.426) (data not shown).

**Table 4:** The proportion of men with CHD who had fathered a child

**Diagnosis**	**Count**^**a**^	**%**	**P-value**^**b**^
**Complex**			
Truncus arteriosus	1	4.0	0.036
Transposition of the great arteries	26	15.5	0.072
Tetralogy of Fallot	29	17.4	0.232
Single ventricle	7	20.6	0.937
**Simple**			
Ventricular septal defect	103	13.2	<0.001
Atrial septal defect	84	15.7	0.002
Endocardial cushion-defect - atrioventricular septal defect	12	11.1	0.011
Triscupid valve defect	10	13.2	0.088
Mitral valve defect	19	14.1	0.044
Pulmonary valve defect	46	17.5	<0.001
Congenital valve stenosis	43	17.6	0.178
Other heart defects	36	13.4	0.002
Congenital aortic insufficiency	58	17.5	0.101
Patent arterial duct	39	11.1	<0.001
Coarctation of the aorta	51	19.2	0.449
Other anomalies of the aorta	17	16.5	0.249
Anomalies of the pulmonary artery	12	13.6	0.085
Anomalies of the great veins	22	18.5	0.478
Non-specified anomalies of the heart or great vessels	69	15.4	<0.001
Total	415	15.4	<0.001

^a^Each person may have more than one CHD diagnosis.

^b^Compared to all other men in the study cohort.

Table 5 shows the delivery-related data of the studied men’s firstborn children. No differences were found in birthweight or gestational length, preterm birth, SGA, LGA, instrumental delivery or caesarean section. Neither was there any difference in the incidence of congenital anomalies in children of men with CHD compared to children of men without the condition.

**Table 5:** **Delivery-related characteristics of the studied men’s firstborn children**^**a**^

	**Complex CHD**		**Simple CHD**		**No CHD**		
	**Count**	**%**	**Count**	**%**	**Count**	**%**	**P-value**
**Birthweight (g) (mean)**^**b**^	3651		3526		3521		
complex vs simple							0.294
complex vs none							0.210
simple vs none							0.982
**Gestational length (weeks) (mean)**^**b**^	39.8		39.3		39.3		
complex vs simple							0.134
complex vs none							0.104
simple vs none							0.996
**Born preterm**^**c**^							0.275
Yes	1	1.9	24	7.5	6894	6.3	
No	53	98.1	297	92.5	102898	93.7	
**Born small-for-gestational-age**^**c**^							0.843
Yes	1	1.9	8	2.5	2253	2.1	
No	53	98.1	306	97.5	105787	97.9	
**Born large-for-gestational age**^**c**^							0.836
Yes	1	1.9	10	3.2	3547	3.3	
No	53	98.1	304	96.8	104493	96.7	
**Twin birth**^**c**^							0.492
Yes	0	0	6	1.9	1471	1.3	
No	54	100	315	98.1	108573	98.7	
**Instrumental delivery**^**c**^							0.753
Yes	6	11.1	37	11.5	11324	10.3	
No	48	88.9	284	88.5	98720	89.7	
**Caesarean section**^**c**^							0.551
Yes	7	13.0	54	16.8	16278	14.8	
No	47	87.0	267	83.2	93766	85.2	
**Congenital anomalies**^**c**^							0.961
Yes	3	5.6	15	4.7	5298	4.8	
No	51	94.4	306	95.3	104746	95.2	

^a^Analysis performed on all 109 928 eligible children.

^b^Analysis of variance corrected by Tukey-Kramer procedure.

^c^Chi-squared test, overall comparisons.

## Discussion

The results of this large national cohort study show that men with CHD are more likely to have been born preterm, SGA or LGA than men without CHD. Men with CHD are also more likely to have been the result of a twin pregnancy and to have been born with a caesarean section. This is in accordance with our earlier findings in women born with a CHD from the same Swedish birth cohort [3]. Men with CHD had a decreased likelihood of becoming a father compared to men without CHD. However, the delivery-related outcomes and the incidence of congenital anomalies in their children did not differ between groups.

The reported risk of CHD in children of men with CHD ranges in different studies between 2.5–3.6% [8,10,11]. However, these studies are based on smaller series of men and women with specific CHD diagnoses. In this study we found no increased risk of congenital anomaly but we were unable to fully investigate the proportion of different CHDs among children with anomalies due to lack of data in the registries. It is important to take into consideration that for a newborn to receive the diagnosis of a CHD in the registries used, the defect has to be diagnosed directly after the delivery before the mother is discharged from the hospital. Many congenital heart defects are not diagnosed until a later stage. This may affect the number of children to men with or without CHD found to have a minor congenital malformation with an underreporting as the consequence. Still, in an earlier study using the same registries and birth cohort we found an increased risk of congenital anomalies when investigating children of mothers with CHD [4].

It is somewhat surprising that men with CHD are born LGA but the reason might be that they are the result of a high-risk pregnancy such as a type 1 diabetes mellitus [25].

The major strength in using data from registries is the large size of collected data and the population-based information free from recall bias. However, register data can involve misclassification problems caused by unrecorded cases and/or incorrect registration of diagnostic codes, and this limitation may have affected the validity of the data used in our study.

Almost 100% of the hospital discharges are registered in the in-patient register. The accuracy of the data is 85-95% depending on the diagnosis [26]. The MBR covers almost 100% of all births in Sweden. The report rate is controlled every year against the births registered in the Birth registry at Statistics Sweden.

To our knowledge this is the first study to investigate the birth characteristics of men with CHD as well as the neonatal outcome of their children.

Registry studies have the advantage that the studied population is large and sometimes as in this study the whole population of a country can be included. This enables large numbers of cases in the groups to be analysed, which is uncommon when rare medical conditions such as CHD are investigated. Since this is a relatively new patient group for obstetricians little is known about how the condition influences the reproductive patterns. The information in registries can describe this and can be used to formulate hypotheses.

There is no risk of selection bias since the whole population is included in the study and investigated. Another advantage of studies of registries is that because the databases were not created for a priori-defined research purposes, there is no opportunity for interviewer bias or ascertainment bias. However, there may be large observed and unobserved differences in patient characteristics that can lead to confounding. In the multivariable logistic regression analysis we tried to control characteristics known to cause confounding in this special study and reduce this.

The major limitation of this study is that malformations of the heart show different patterns, which cause a problem of inhomogenity among the studied individuals. Every heart malformation is different. Hence, it is not possible to have homogeneous groups. This makes it difficult to draw valid conclusions and generalizations about the different subgroups of CHD. To try to make results more useful, the diagnoses were divided into subgroups. Another major limitation is the relative lack of clinical data concerning men with CHD.

It is still necessary to design studies that try to describe the outcomes of pregnancy and birth of the children of these parents since CHD is the most common congenital abnormality. The incidence of surviving individuals with CHD is growing and this means that an ever increasing number of men and women with CHD want to become parents.

## Conclusions

This large national cohort study has shown that men with CHD are more likely to have been born preterm, SGA or LGA but the increased risk does not repeat itself in the next generation. Neither was there any increased risk of congenital malformations in offspring to men with CHD in this study. The result is of importance for cardiologists, obstetricians or others who give genetic and pre-conception counselling to men and women with CHD.

## Competing interests

The authors do not report any conflicts of interest.

## Authors’ contributions

KK and AJ had the original research idea. All authors planned the study. KK and MB analysed the data and drafted the paper. All authors contributed to the interpretation of the data, revisions and gave input at all stages of the study. All authors have approved to the final version of the manuscript.

## Pre-publication history

The pre-publication history for this paper can be accessed here:

http://www.biomedcentral.com/1471-2393/14/187/prepub

## References

[B1] Harris IS Management of pregnancy in patients with congenital heart disease Prog Cardiovasc Dis 2011 53 305 311 10.1016/j.pcad.2010.08.001 21295672 PMC3052938

[B2] Uebing A Gatzoulis MA von Kaisenberg C Kramer H-H Strauss A Congenital heart disease in pregnancy Dtsch Arztebl Int 2008 105 347 354 19629245 10.3238/arztebl.2008.0347 PMC2696871

[B3] Josefsson A Kernell K Nielsen NE Bladh M Sydsjö G Reproductive patterns and pregnancy outcomes in women with congenital heart disease – a Swedish population-based study Acta Obstet Gynecol Scand 2011 90 659 665 10.1111/j.1600-0412.2011.01100.x 21314820

[B4] Siu SC Sermer M Harrison DA Grigoriadis E Liu G Sorensen S Smallhorn JF Farine D Amankwah KS Spears JC Colman JM Risk and Predictors for pregnancy-related complications in women with heart disease Circulation 1997 96 2789 2794 10.1161/01.CIR.96.9.2789 9386139

[B5] Drenthen W Pieper PG Roos-Hesselink JW van Lottum WA Voors AA Mulder BJ van Dijk AP Vliegen HW Yap SC Moons P Ebels T van Veldhuisen DJ ZAHARA Investigators Outcome of pregnancy in women with congenital heart disease: a literature review J Am Coll Cardiol 2007 49 2303 2311 10.1016/j.jacc.2007.03.027 17572244

[B6] Siu SC Sermer M Colman JM Alvarez AN Mercier LA Morton BC Kells CM Bergin ML Kiess MC Marcotte F Taylor DA Gordon EP Spears JC Tam JW Amankwah KS Smallhorn JF Farine D Sorensen S Cardiac Disease in Pregnancy (CARPREG) Investigators Prospective multicenter study of pregnancy outcomes in women with heart disease Circulation 2001 104 515 521 10.1161/hc3001.093437 11479246

[B7] Vriend JW Drenthen W Pieper PG Roos-Hesselink JW Zwinderman AH van Veldhuisen DJ Mulder BJ Outcome of pregnacy in patients after repair of aortic coarctation Eur Heart J 2005 26 2173 2178 10.1093/eurheartj/ehi338 15946957

[B8] Nora JJ Nora AH Update on counseling the family with a first degree relative with a congenital heart defect Am J Med Genet 1988 29 137 142 10.1002/ajmg.1320290117 3344765

[B9] Romano-Zelekha O Hirsch R Blieden L Green M Shohat T The risk for congenital heart defects in offspring of individuals with congenital heart defects Clin Genet 2001 59 325 329 11359463 10.1034/j.1399-0004.2001.590505.x

[B10] Connolly HM Warnes CA Ebstein’s anomaly: outcome of pregnancy J Am Coll Cardiol 1994 23 1194 1198 10.1016/0735-1097(94)90610-6 8144788

[B11] Driscoll DJ Michels VV Gersony WM Hayes CJ Keane JF Kidd L Pieroni DR Rings LJ Wolfe RR Weidman WH Occurrence risk for congenital heart defect in relatives of patients with aortic stenosis, pulmonary stenosis, or ventricular septal defect Circulation 1993 87 114 120 8425317

[B12] Cnattingius S Ericson A Gunnarskog J Kallen B A quality study of a medical birth registry Scand J Soc Med 1990 18 143 148 2367825 10.1177/140349489001800209

[B13] Centre for Epidemiology, National Board of Health and Welfare The Swedish Medical Birth Registry; A Summary of Content and Quality (Article no. 2003-112-3) 2003 Stockholm, Sweden http://www.socialstyrelsen.se/publikationer2003/2003-112-3

[B14] Statistics Sweden A new Total Population Registry System. More Possibilities and Better Quality. (Serial no. 2002:2) 2002 Örebro, Sweden: Statistics Sweden

[B15] Centre for Epidemiology, National Board of Health and Welfare In-Patient Diseases in Sweden 1987-2001 (Article no. 2003-42-8) 2003 Stockholm, Sweden: National Board of Health and Welfare http://www.socialstyrelsen.se/publikationer2003/2003-42-8

[B16] Centre for Epidemiology, National Board of Health and Welfare The Swedish Version of 8th Revision of WHO’s International Classification of Diseases 1968 Stockholm, Sweden: National Board of Health and Welfare http://www.socialstyrelsen.se/klassificeringochkoder/diagnoskoder/historiska-klassifikationer

[B17] Centre for Epidemiology, National Board of Health and Welfare The Swedish Version of 9th Revision of WHO’s International Classification of Diseases 1987 Stockholm, Sweden: National Board of Health and Welfare http://www.socialstyrelsen.se/klassificeringochkoder/diagnoskoder/historiska-klassifikationer

[B18] Centre for Epidemiology, National Board of Health and Welfare The Swedish Version of 10th Revision of WHO’s International Classification of Disease 1997 Stockholm, Sweden: National Board of Health and Welfare http://www.socialstyrelsen.se/klassificeringochkoder/diagnoskoder

[B19] Centre of Epidemiology, National Board of Health and Welfare Causes of Death 2001 2003 Stockholm, Sweden: National Board of Health and Welfare http://www.socialstyrelsen.se/register/dodsorsaksregistret

[B20] Statistics Sweden Multi-Generation Registry 2002; a Description of Contents and Quality (Serial no. 2003:5) 2003 Örebro, Sweden: Statistics Sweden

[B21] Statistics Sweden Population and Housing Census 1970 (SOS). Part 12. Report on the Planning and Processing of the Population and Housing Census 1970 1974 Stockholm, Sweden: National Central Bureau of Statistics

[B22] Statistics Sweden Educational Attainment of the Population 2002. (Publication no. UF0506) 2003 Örebro, Sweden: Statistics Sweden http://www.scb.se/en_/

[B23] Statistics Sweden Population and Housing Census 1970. Part 13. Economic Activity and Education. Definitions, Comparability, Development, etc 1975 Stockholm, Sweden: National Central Bureau of Statistics

[B24] Marsal K Persson PH Larsen T Lilja H Selbing A Sultan B Intrauterine growth curves based on ultrasonically estimated foetal weights Acta Paediatr 1996 85 843 848 10.1111/j.1651-2227.1996.tb14164.x 8819552

[B25] Rosenn BM Miodovnik M Holcberg G Khoury JC Siddiqi TA Hyperglycemia: The price of intensive insulin therapy for pregnant women with insulin-dependent diabetes mellitus Obstet Gynecol 1995 85 417 422 10.1016/0029-7844(94)00415-A 7862383

[B26] Ludvigsson JF Andersson E Ekbom A Feychting M Kim JL Reuterwall C Heurgren M Olausson PO External review and validation of the Swedish national inpatient register BMC Public Health 2011 11 450 10.1186/1471-2458-11-450 21658213 PMC3142234

